# Supplementary material for: Actinidia eriantha Benth. Root as a New Phytomedicine Inhibits Non-Small Cell Lung Cancer by Regulating·TGF-β/FOXO/mTOR
Source: Int J Mol Sci. 2025 Sep 14;26(18):8957. doi: 10.3390/ijms26188957 (PMC12469755; doi:10.3390/ijms26188957)
Supplement: Supplementary file 1 [file ijms-26-08957-s001.zip › ijms-3853096-supplementary.pdf]

Actinidia Eriantha Benth. Root as a New Phytomedicine Inhibits Non-Small Cell Lung Cancer by Regulating· TGF-β/FOXO/mTOR

Xuan Zhang[1,2], Qiyao Xiao[1], Haoran Chen[1], Shaoming Yang[3], Qingli Li[3], Lihua Peng[1,2]*

[1] College of Pharmaceutical Sciences, Zhejiang University, Hangzhou 310058, Zhejiang, PR China.

[2] Jinhua Institute of Zhejiang University, Jinhua 321299, Zhejiang, PR China.

[3] Zhejiang Longquan Zhengda Biological Technology Co., Ltd, Longquan 323700, Zhejiang, PR China.

*Address correspondence to

Lihua Peng, Ph.D., Professor.

College of Pharmaceutical Sciences, Zhejiang University, 866# Yuhangtang Road, Hangzhou, 310058, P.R. China.

Email: lhpeng@zju.edu.cn

Tel/Fax: +86-571-88981231

Table S1. Detailed LCMS/MS Characterization of the Small Molecules

Number	Name	RT	m/z
1	L-(+)-lactic acid	5.999	89.0228167
2	Anserine	9.014	239.1143671
3	Creatinine	7.984	112.0502094
4	Linoleic acid	0.78	279.2325795
5	1-hydroxy-2-naphthoic acid	0.455	187.0417624
6	Palmitic acid	0.785	255.2324813
7	Sarcosine	7.938	88.03886268
8	Mefenamic acid	9.014	240.1148801
9	Oleic acid	0.78	281.2478074
10	Octadecanoic acid	0.771	283.2637515
11	Inosine 5'-monophosphate	9.584	347.0392292
12	Hydroquinidine	0.588	325.183662
13	Hypoxanthine	4.964	135.0299595
14	Creatine	7.938	130.0608601
15	Inosine	5.785	267.073088
16	Uridine	4.819	243.0616501
17	Uracil	2.91	111.0185947
18	Caffeic acid	10.055	178.8833018
19	Pantothenic acid	6.789	218.1026848
20	Leucine	6.727	130.086055
21	Terephthalic acid	7.927	165.0183246
22	D-arabinonic acid	7.984	165.039303
23	Histamine	9.133	110.0707719
24	Glucose	7.237	179.0550501
25	Gallic acid	0.736	169.0165469
26	Allyl isothiocyanate	5.038	98.02328959
27	Dihydrokaempferol	4.843	287.0435743
28	Flavone	6.639	221.0671863
29	Linolenic acid	0.785	277.2168849

Top ten bioactive compounds highlighted in red.
